# Supplementary material for: End-to-end design of multicolor scintillators for enhanced energy resolution in X-ray imaging
Source: Light Sci Appl. 2025 Apr 11;14:158. doi: 10.1038/s41377-025-01836-8 (PMC11985908; doi:10.1038/s41377-025-01836-8)
Supplement: Supplementary file 1 — Supplementary Information for End-to-end design of multicolor scintillators for enhanced energy resolution in X-ray imaging [file 41377_2025_1836_MOESM1_ESM.pdf]

Supplementary Information for End-to-end design of multicolor scintillators for enhanced energy resolution in X-ray imaging

Seokhwan Min[1,2], Seou Choi[1], Simo Pajovic[3], Sachin Vaidya[4], Nicholas Rivera[5], Shanhui Fan[6], Marin Soljačić[1,4], Charles Roques-Carmes[1,6*]

[1]Research Laboratory of Electronics, Massachusetts Institute of Technology, 77 Massachusetts Ave., Cambridge, 02139, MA, USA.
[2]Department of Material Science and Engineering, Korea Advanced Institute of Science and Technology, 291 Daehak-ro, Daejeon, 34141, Daejeon, Republic of Korea.
[3]Department of Mechanical Engineering, Massachusetts Institute of Technology, 77 Massachusetts Ave., Cambridge, 02139, MA, USA.
[4]Department of Physics, Massachusetts Institute of Technology, 77 Massachusetts Ave., Cambridge, 02139, MA, USA.
[5]Department of Physics, Harvard University, Massachusetts Hall, Cambridge, 02138, MA, USA.
[6]E. L. Ginzton Laboratories, Stanford University, 450 Jane Stanford Way, Stanford, 94305-2048, CA, USA.

*Corresponding author(s). E-mail(s): chrc@stanford.edu; Tel. +1-650-723-2300
Contributing authors: seokhwan.min@kaist.ac.kr; seouc130@mit.edu; pajovics@mit.edu; svaidya1@mit.edu; nrivera@fas.harvard.edu; shanhui@stanford.edu; soljacic@mit.edu;

S1. Energy reconstruction with single-layer scintillators

Single-layer scintillators have energy-dependent X-ray absorption profiles (Fig. S1a, Fig. S2a) and show the resulting spatial separation of different energies (Fig. S1b, Fig. S2b). To utilize these properties for energy reconstruction, the single-layer scintillators must be used with our postprocessing algorithm that identifies scintillation clusters and reconstructs the X-ray energy based on the cluster radius.

Even so, single-layer scintillators have two major disadvantages: (1) K-edges can disrupt the spatial separation of energies and degrade the energy reconstruction accuracy (Fig. S2) and; (2) thick scintillators are required to achieve better spatial separation of energies, which compromises the X-ray image resolution (Fig. S3).

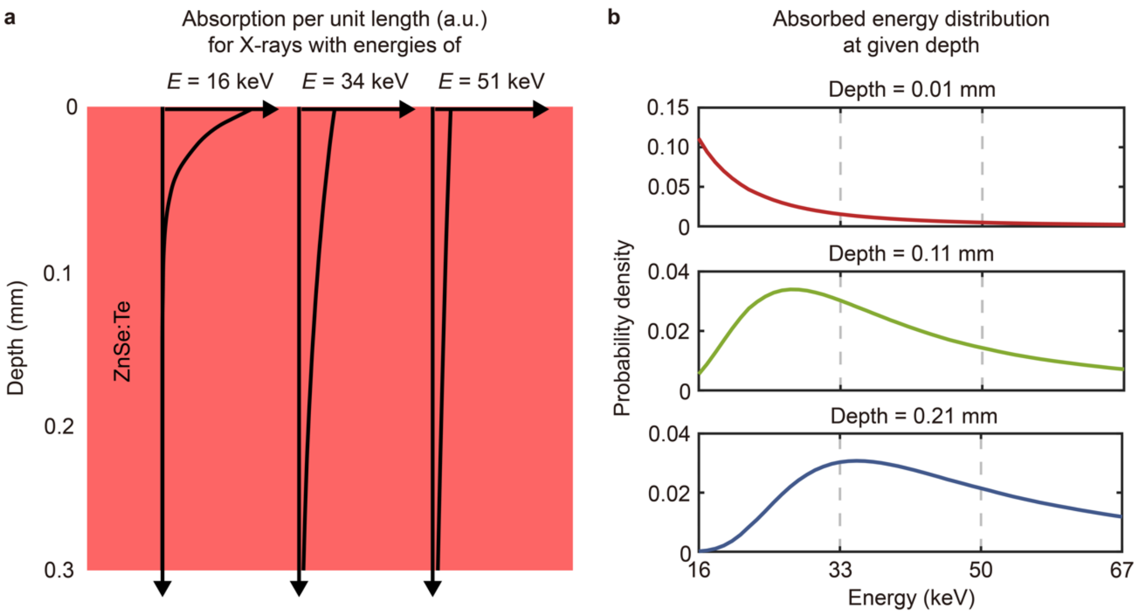

Fig. S1 Energy discrimination using a single-color scintillator without an absorption edge. **a** Absorption per unit length along the X-ray propagation path for different X-ray energies. The scintillator is 0.3 mm thick. **b** Absorbed energy distributions at specific depths along the scintillator.

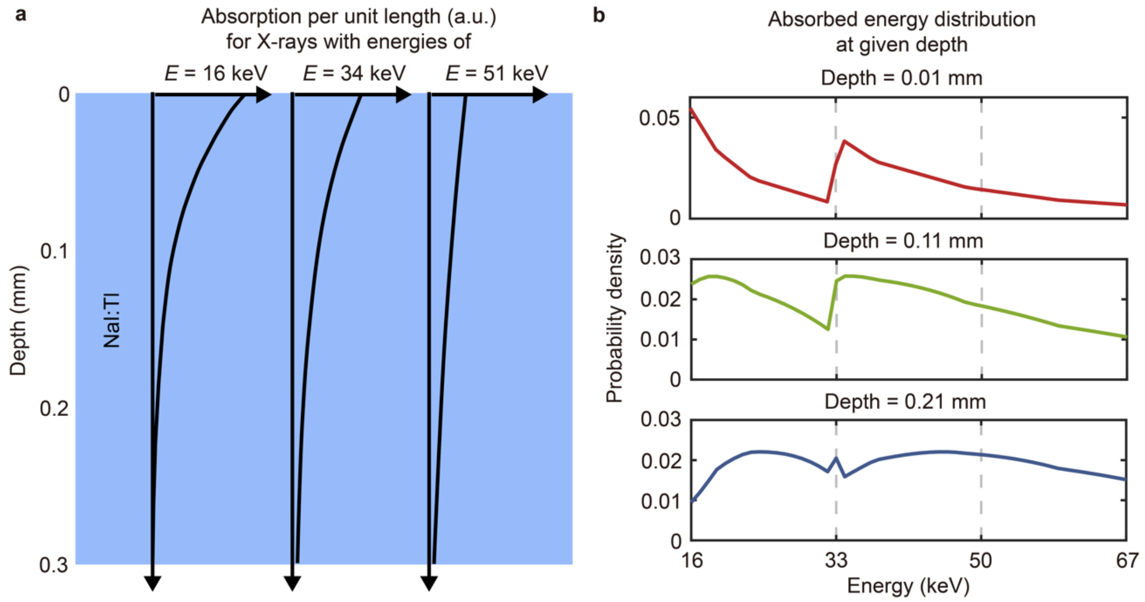

Fig. S2 Energy discrimination using a single-color scintillator with an absorption edge. a Absorption per unit length along the X-ray propagation path for different X-ray energies. The scintillator is 0.3 mm thick. **b** Absorbed energy distributions at specific depths along the scintillator.

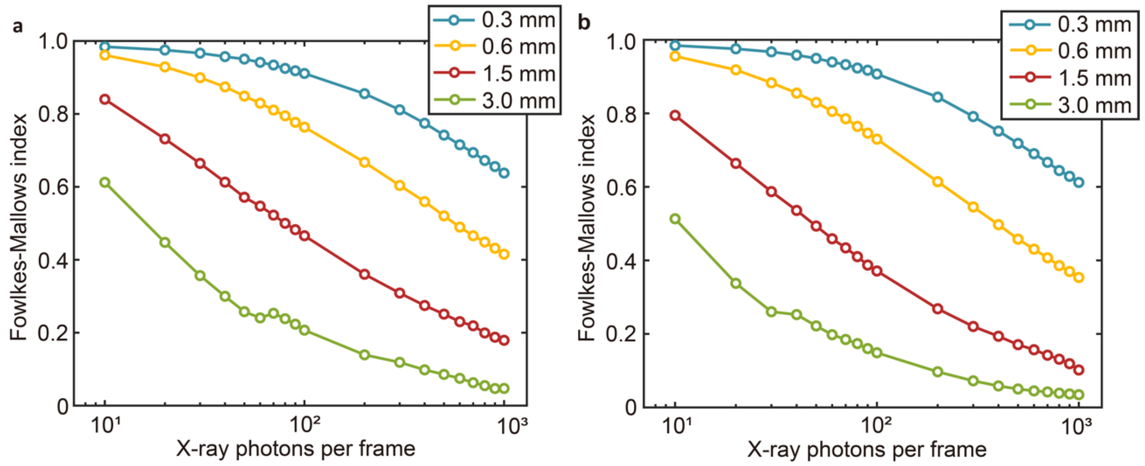

Fig. S3 Fowlkes-Mallows index for single-color scintillators. a ZnSe:Te **b** NaI:Tl

S2. Necessity of Both Color and Cluster Radius Information for Energy Reconstruction

Due to the drastic variation of the absorption profile with energy in the multicolor scintillator, knowing the X-ray absorption depth corresponding to each scintillation spot allows the postprocessing algorithm to make “educated guesses” for the X-ray energy bin. In real-world implementations, we only have access to the scintillation spots that are measured by RGB detectors. The color and radius information extracted from the scintillation spots are indirect indicators of the absorption depth. Due to uncertainties associated with each quantity, they must be used in conjunction to estimate the absorption depth for maximal energy reconstruction accuracy. Comparison of the energy accuracy for a 0.3 mm-thick multicolor scintillator indicates that using both color and radius information (48.3%) yields better energy accuracy than using only color (43.1%) or only radius (45.0%) information.

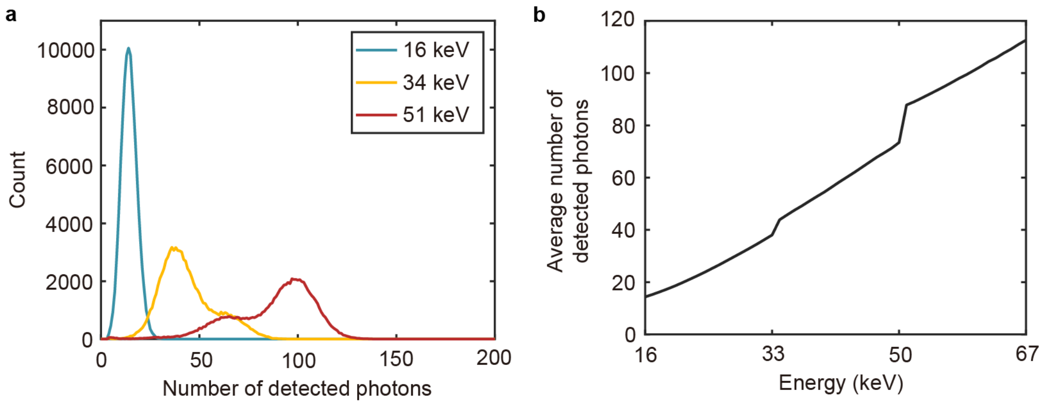

Figure S4. **a** The distribution of the number of scintillation photons that reach the detector for selected X-ray energies. **b** The average number of detected photons as a function of X-ray energy.

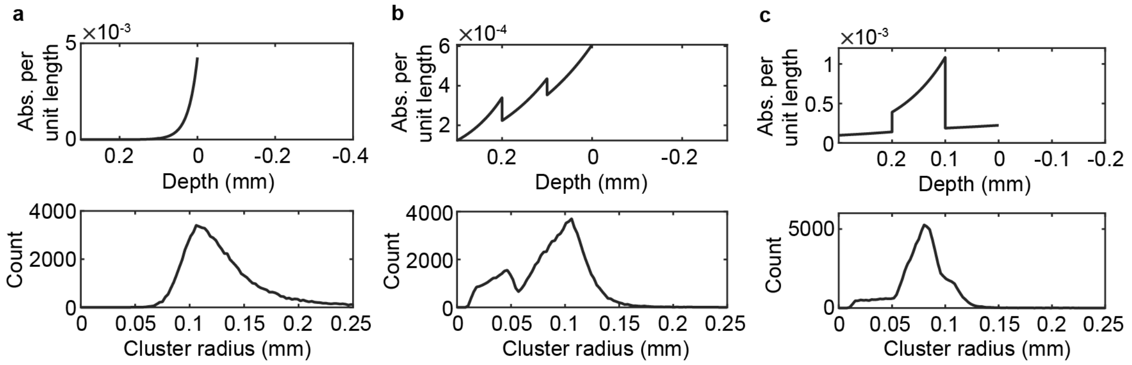

Figure S5. The absorption per unit length (top) and scintillation cluster radius distribution (bottom) due to incident X-rays with energies of **a** 16 keV, **b** 34 keV, and **c** 51 keV onto a 0.3 mm-thick multicolor scintillator.

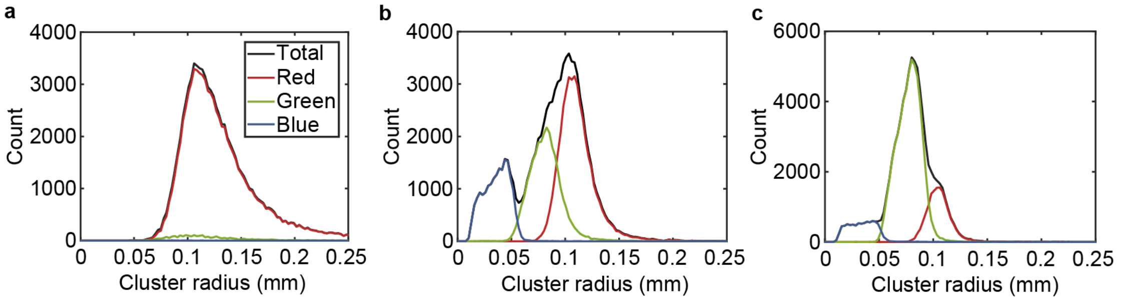

Figure S6. The scintillation cluster radius distribution due to incident X-rays with energies of **a** 16 keV, **b** 34 keV, and **c** 51 keV onto a 0.3 mm-thick multicolor scintillator. The total distributions are in black, which are decomposed into contributions from the ZnSe:Te (red), Gadox:Tb (green), and NaI:Tl (blue) scintillators.

S3. Optimal Layer Thicknesses for the Multicolor and Single-Layer Scintillators

In this section, we describe the detailed optimization procedure that was used to design the multicolor and single-layer scintillators. The optimization objective was to maximize the energy reconstruction accuracy η, which was computed using a surrogate model. The surrogate model was used because the full Monte Carlo simulator GEANT4 currently does not allow direct gradient computation and is also too time-consuming to be run numerous times as part of an optimization routine. The principle behind the energy reconstruction capability of the multicolor scintillator is that the scintillation spot color and size indicate the depth at which the corresponding X-ray was absorbed. Due to the K-edges of Gadox:Tb and NaI:Tl, the absorption profile of each X-ray energy bin has a strong depth dependence. Therefore, the X-ray energy can be estimated from the measured absorption depth. As shown in Fig. S7, the surrogate model simplifies this process by assuming that the X-ray absorption depth can be measured directly (instead of indirectly from the scintillation spot color and size).

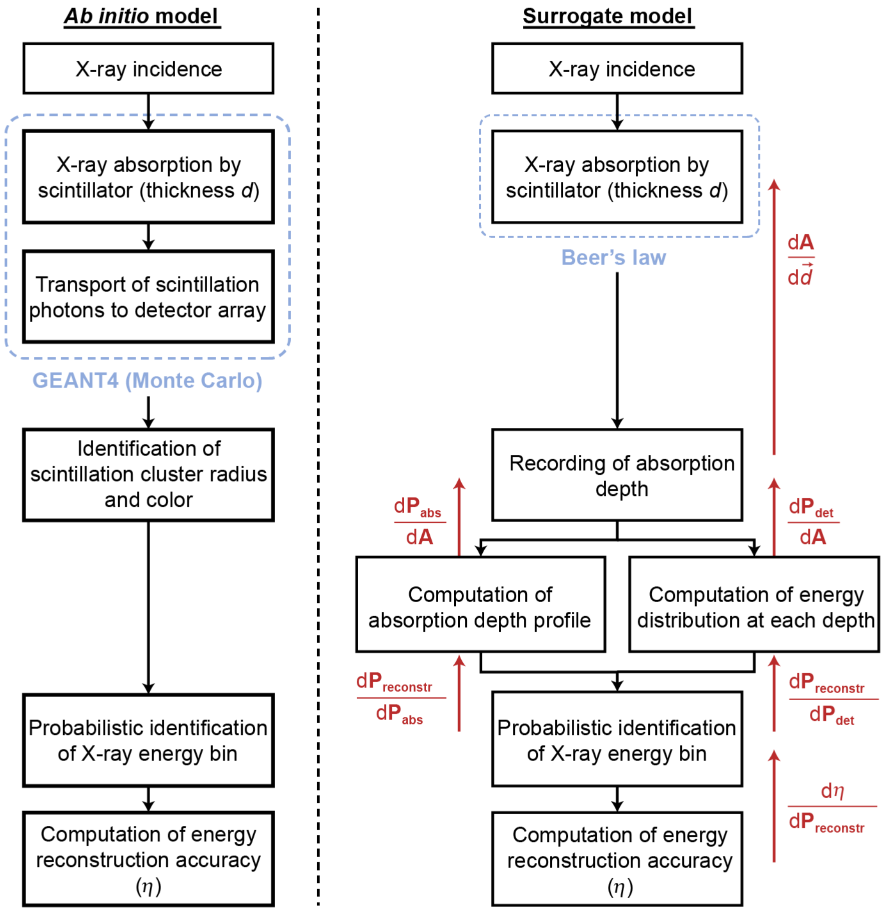

Figure S7. A schematic comparing the *ab initio* GEANT4-based scintillation-reconstruction model and the Beer's law-based surrogate model. The surrogate model assumes a hypothetical detector that can directly measure the X-ray absorption depth.

Under this assumption, the entire X-ray imaging process can be divided into two parts: (1) the action of the multicolor scintillator on the incoming X-ray (i.e. energy-dependent absorption of the X-ray photons; mapping of the ground truth energy to absorption depth) and (2) the action of the postprocessing algorithm (i.e. depth-dependent classification of the resulting scintillation emission to each energy bin; mapping of the absorption depth to reconstructed energy). Therefore, the action of the entire framework (multicolor scintillator + postprocessor) can be summarized as a mapping of the (ground truth) incident energy to the reconstructed energy.

In the following discussion, we discretize the energies (E) and depths (z) into N_E and N_z points respectively, in order to better represent the numerical computation process used in the surrogate model (i.e. a discretized formulation of the continuous form presented in the section "Working principles of the multicolor scintillator" of the main text). In order to model the actions of the scintillator and the postprocessor, the energy-dependent X-ray absorption profile of the scintillator must be computed. This can be collected into an $N_z \times N_E$ matrix $\mathbf{A}$:

$$\mathbf{A} = \boldsymbol{\mu} \odot \exp[\mathrm{diag}(\vec{z})\boldsymbol{\mu}] = \begin{bmatrix} \vec{A}(E_1) & \cdots & \vec{A}(E_{N_E}) \end{bmatrix} = \begin{bmatrix} \vec{A}(z_1)^{\mathrm{T}} \\ \vdots \\ \vec{A}(z_{N_z})^{\mathrm{T}} \end{bmatrix}$$

where $\boldsymbol{\mu}$ is an $N_z \times N_E$ matrix of the depth and energy-dependent linear attenuation coefficient of the scintillator stack, and the exponentiation is done elementwise. The symbol $\odot$ represents the Hadamard product. $A(E_i)$ is the absorption profile of X-rays at energy E_i and $A(z_i)$ is the energy distribution of X-rays absorbed at depth z_i.

Then, the action of the scintillator on the incident X-ray spectrum is given by:

$$\vec{I}_z = \mathbf{P}_{\mathrm{abs}} \vec{I}_E$$

where $\vec{I}_E$ is the discretized incident X-ray energy spectrum and $\vec{I}_z$ is the discretized depth distribution of absorbed X-rays in the scintillator. $\mathbf{P}_{\mathrm{abs}}$ is given as the matrix:

$$\mathbf{P}_{\mathrm{abs}} = \begin{bmatrix} \frac{\vec{A}(E_1)}{\|\vec{A}(E_1)\|_1} & \cdots & \frac{\vec{A}(E_{N_E})}{\|\vec{A}(E_{N_E})\|_1} \end{bmatrix}$$

such that each column is a probability mass function describing the absorption profile of each incident energy. Similarly, the action of the postprocessor on the detected absorption depth distribution is given by:

$$\mathbf{P}_{\mathrm{det}} = \begin{bmatrix} \frac{\vec{A}(z_1)}{\|\vec{A}(z_1)\|_1} & \cdots & \frac{\vec{A}(z_{N_z})}{\|\vec{A}(z_{N_z})\|_1} \end{bmatrix}$$

such that each column is a probability mass function describing the known energy distribution of X-rays absorbed at each depth. Accordingly, the action of the entire framework is a cascade of the actions of the scintillator and the postprocessor:

$$\mathbf{P}_{\mathrm{reconstr}} = \mathbf{P}_{\mathrm{det}} \mathbf{P}_{\mathrm{abs}}$$

where $\mathbf{P}_{\text{reconstr}}$ is an $N_E \times N_E$ matrix that maps the incident energies to the reconstructed energies (Fig. S8). In practice, we only care about the accurate mapping of energies from each ***energy bin*** to itself. Therefore, $\mathbf{P}_{\text{reconstr}}$ is divided into 3 × 3 blocks whose elements are summed up, resulting in a 3 × 3 confusion matrix. The trace of the confusion matrix divided by 3 represents the average energy reconstruction accuracy of the entire framework, and this was used as the optimization figure of merit. Note that the confusion matrix of an ideal scintillator with perfect energy accuracy will be the identity matrix. Although this surrogate model describes the relationship between the incident and reconstructed X-ray spectra (or energy bin distribution) as a whole, it is still based on the behavior of single photons according to specific probability distributions, akin to the *ab initio* Monte Carlo simulation.

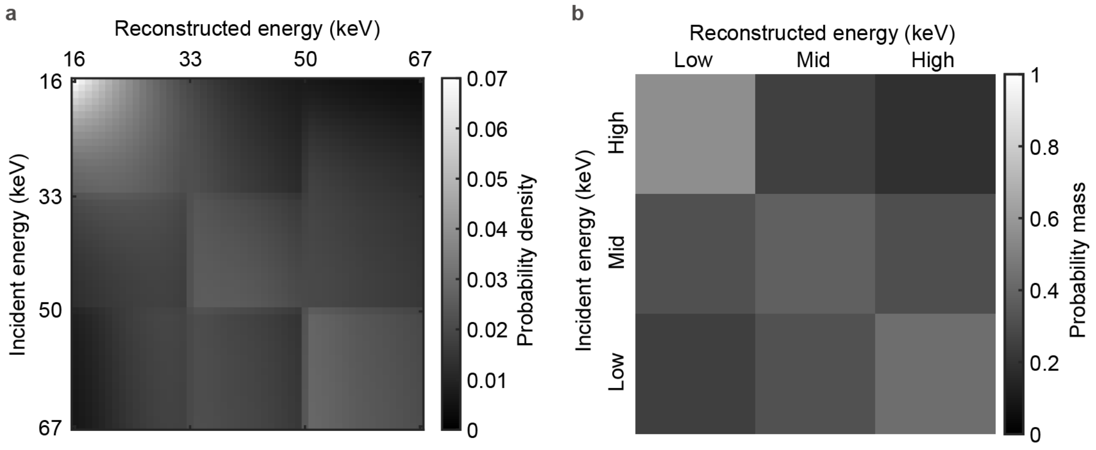

Figure S8. **Confusion matrices. a** Confusion matrix for discretized energies. **b** Confusion matrix for energy bins.

Using the above surrogate model, we optimized the layer thicknesses of the multicolor scintillator using L-BFGS-B. Several optimization runs were done with the following upper bounds on the individual layer thicknesses: 0.1, 0.2, 0.5, and 1.0 mm. For comparison, ZnSe:Te and NaI:Tl single-layer scintillators were also optimized, but with increased upper bounds (i.e. 0.3, 0.6, 1.5, 3.0 mm) to keep the overall thickness equal to that of the multicolor scintillators. The energy accuracies (according to the surrogate model and the *ab initio* simulation) of the optimized multicolor scintillators are plotted

alongside those of single-layer scintillators in Fig. S9. The optimized thicknesses are listed in Table S1. In all cases except for the design with the 1.0 mm upper bound, the larger the thickness, the better the accuracy. This is because thicker scintillators can function better as energy filters for the lower layers, which improves the energy accuracy.

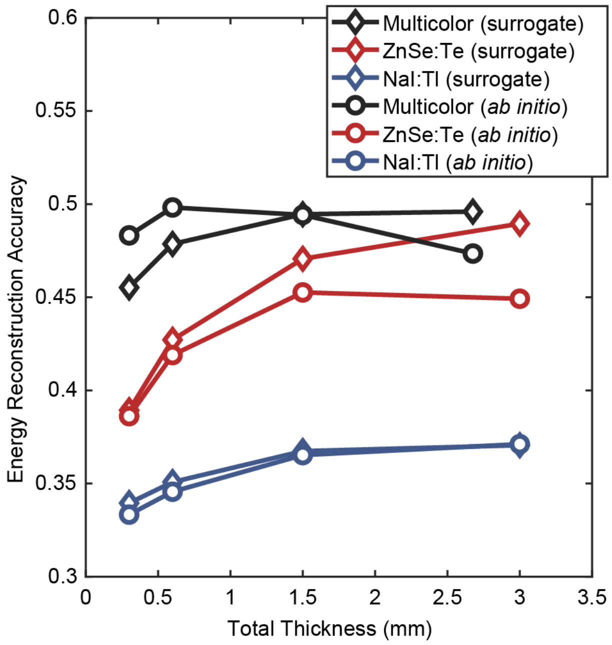

Figure S9. Energy reconstruction accuracies for the multicolor, ZnSe:Te, and NaI:Tl scintillators with optimized thicknesses. The accuracies shown were computed using both the *ab initio* simulation and the simplified surrogate model for the scintillation-reconstruction process.

Table S1 Optimized scintillator thicknesses under different thickness upper bounds.

Type		Layer Thickness Upper Bound (mm)			
		0.1 (3-layer)<br>0.3 (single)	0.2 (3-layer)<br>0.6 (single)	0.5 (3-layer)<br>1.5 (single)	1.0 (3-layer)<br>3.0 (single)
**Multicolor**	**ZnSe:Te**	0.100	0.200	0.500	0.776
	**Gadox:Tb**	0.100	0.200	0.500	0.984
	**NaI:Tl**	0.100	0.200	0.500	0.915
**ZnSe:Te**		0.300	0.600	1.500	3.000
**NaI:Tl**		0.300	0.600	1.500	3.000

We also note that the same surrogate model was used for the computation of the energy reconstruction accuracy upper bound for fixed thicknesses and variable linear attenuation coefficients (see section "Energy reconstruction accuracy bounds" of the main text). Fig. S10 shows plots of the linear attenuation coefficient model parameters that were referred to when setting the parameter bounds.

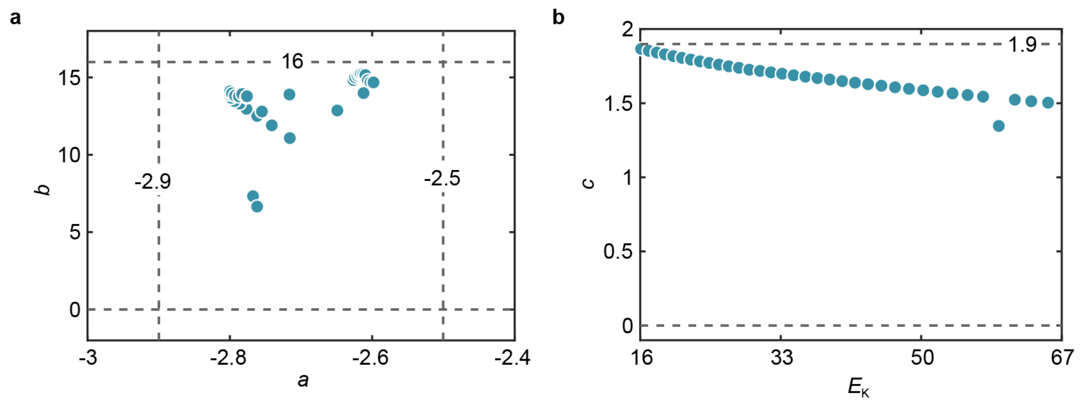

Fig. S10 a-b Values of the linear attenuation coefficient model parameters for existing elements and scintillators.

S4. Robustness of the Multicolor Scintillator Energy Reconstruction Framework to the Scintillator Emission Spectra

Spectral overlap of scintillator emission can cause scintillation spots to have the "wrong" dominant color (e.g. dominantly green scintillation spot from ZnSe:Te). In typical energy-resolving schemes that rely on emission color, this can cause significant degradation of the energy resolution. On the other hand, even though our method does rely on color information to reconstruct the incoming energy, it is designed to be more robust to the spectral overlap of the scintillator emission. This is primarily because our approach uses ***both color and size*** information to take into account the overlap of the scintillation spectra.

For example, the absorption of intermediate-energy X-rays by the red-emitting ZnSe:Te may result in some scintillation spots that are dominantly green instead of red, because of the partial spectral overlap between ZnSe:Te and Gadox:Tb. Nevertheless, the green spots from ZnSe:Te will generally be larger than the green spots from the green-emitting Gadox:Tb. The conditional probability reflects this information and the postprocessor assigns an increased proportion of green spots with large radii to the intermediate energy bin.

Figs. S11-13 show simulation results comparing cases with different amounts of overlap between the scintillation spectra. The overlap was varied in two different ways: (1) by artificially "stretching" the emission spectra of all scintillators by a factor of 2 (Fig. S11b) and; (2) by artificially red-shifting the emission of NaI:Tl and blue-shifting the emission of ZnSe:Te by 50 nm (Fig. S11c). Fig. S12 shows the color and cluster radius distribution of scintillation spots resulting from 16, 42, and 67 keV X-rays. Fig. S13 shows the conditional probability for the X-ray energy for each color given cluster radii of 0.036, 0.083, and 0.109 mm.

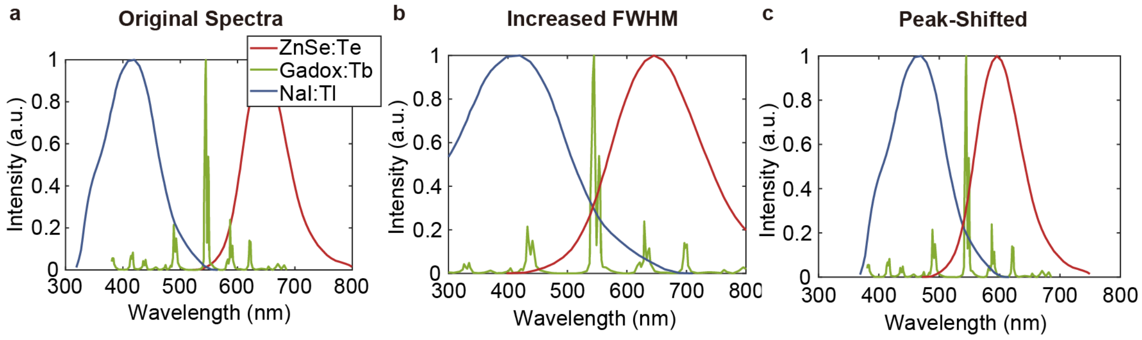

Figure S11. **a** Original scintillation emission spectra. **b** Scintillation spectra with twice the FWHM of the original. **c** Peak-shifted scintillation spectra. The ZnSe:Te spectrum was blue-shifted by 50 nm and the NaI:Tl spectrum was red-shifted by 50 nm.

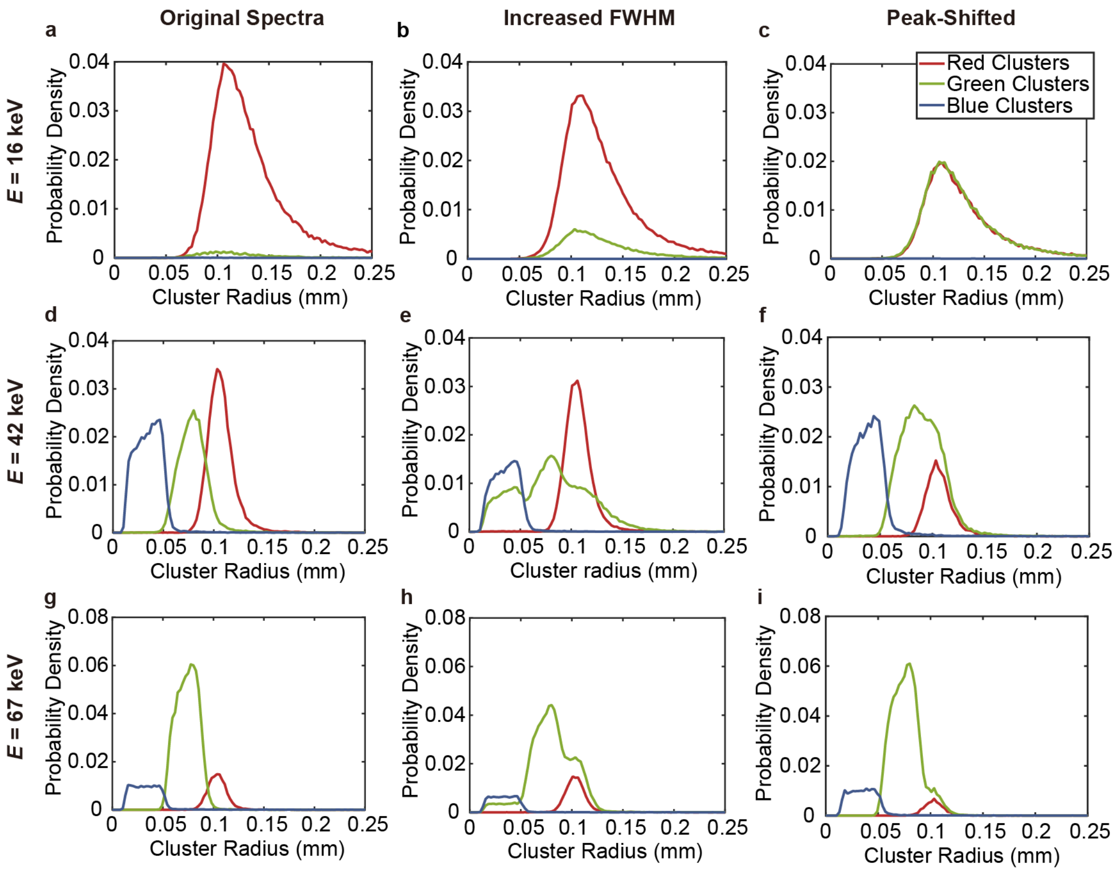

Figure S12. Distribution of the cluster radii of scintillation photons generated by X-rays with energies of 16 (top), 42 (middle), and 67 keV (bottom). Simulations were done using the original (left), stretched (middle), and shifted (right) scintillation spectra.

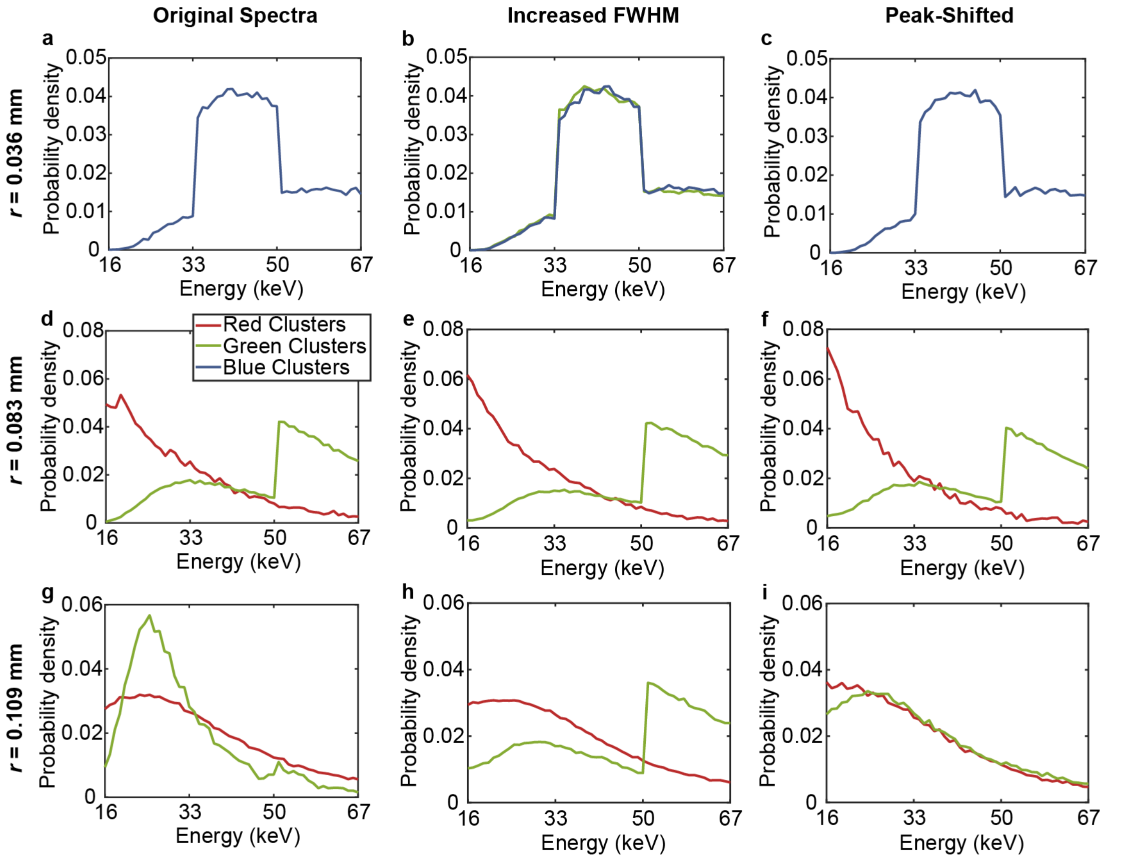

Figure S13. Conditional probabilities for the X-ray energy for red, green, and blue clusters for radii of 0.036 (top), 0.083 (middle), and 0.109 mm (bottom). The probabilities were computed from data collected from simulations with the original (left), stretched (middle), and shifted (right) scintillation spectra. Some color-radius combinations are omitted due to a lack of observed samples (i.e. red clusters with near 0.036 mm radii are unlikely to occur).

When overlap is small, the radius distributions of the scintillation spots from each scintillator are well-separated (Fig. S12d, g). Upon overlap increase, significant portions of the emission spots from ZnSe:Te and NaI:Tl start to become green (i.e. there are increased numbers of green clusters with large and small radii) (Fig. S12e, h). For the peak-shifted case, the emission color from NaI:Tl is mostly unaffected (although its absolute color changes, it is still dominantly blue), but a much more significant amount of emission spots from ZnSe:Te becomes dominantly green (Fig. S12c, f, i).

These changes are taken into account by the conditional probability. For the case with increased FWHM, green clusters with small radii are treated just like blue clusters with small radii and are classified into the intermediate-energy bin (and the high-energy bin to a lesser extent) (Fig. S13b). Green clusters with large radii are classified mainly into the high-energy bin (Fig. S13h). For the peak-shifted case, green clusters with large radii are treated the same as red clusters with large radii and classified mainly into the low and intermediate energy bins. The changes in the classification probabilities are especially noticeable for large radii (Fig. S13g, h, i).

Finally, the energy reconstruction accuracy was computed for each simulation condition. Even after a twofold increase in FWHM and 50 nm peak shifts, the accuracy remains at 47.2% and 47.7% respectively, which are 97.7% and 98.7% of the original value (48.3%). More importantly, the accuracies are still significantly higher than those of single-layer ZnSe:Te and NaI:Tl scintillators (38.6% and 33.3%). These results indicate that the energy reconstruction accuracy is relatively robust to emission spectra overlap under our reconstruction framework.

S5. Spatial Resolution of the Multicolor Scintillator

Fig. S14a shows the spot size distribution for several X-ray energies for the 0.3 mm-thick multicolor scintillator. The average spot size has been plotted in Fig. S14b as a function of the X-ray energy. The average spot size ranges between 0.07 and 0.133 mm, which is within the range of spatial resolutions encountered in typical X-ray radiography and mammography (5-15 line pairs per millimeter or 0.067-0.2 mm resolution[1]).

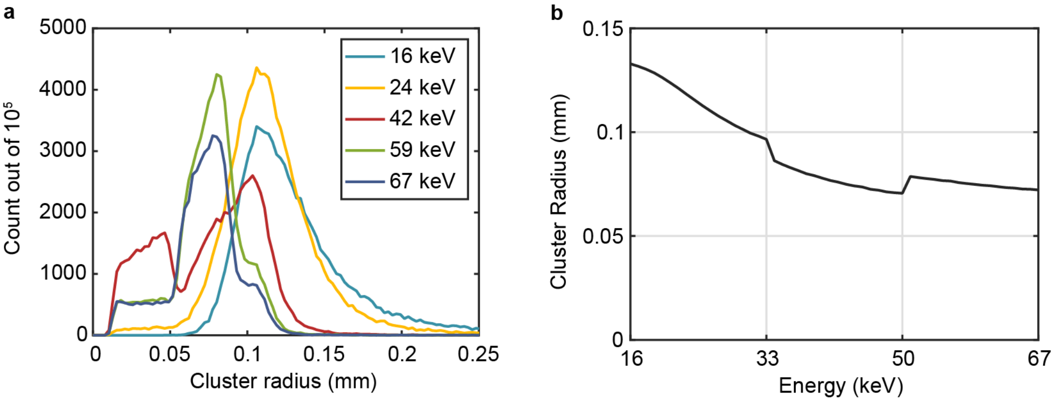

Figure S14. **a** Cluster radius distribution for selected X-ray energies for the 0.3 mm-thick multicolor scintillator. **b** Average cluster radius as a function of the incident energy.

S6. Medical Phantom Imaging

Fig. S15a shows the effective linear attenuation coefficients of each cylindrical region in the medical phantom. The effective linear attenuation coefficients were computed as:

$$\mu_{\text{eff}}(E) = \frac{1}{d}\int_{z=0}^{z=d} \mu(z, E)\mathrm{d}z$$

where z is the coordinate along the depth of the phantom, E is the energy, and d is the overall thickness of the phantom. Fig. S15b shows the phantom's energy-integrated X-ray transmission where it is clear that the cylindrical regions cannot be distinguished from image contrast alone.

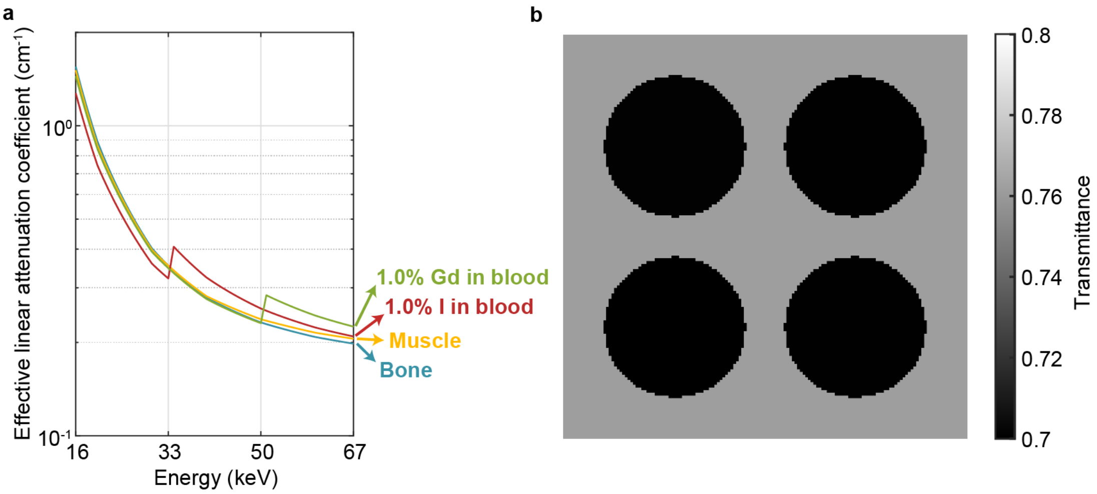

Fig. S15 Simulated performance of energy-integrating detector for the medical phantom. a The effective attenuation coefficient along the phantom depth in each cylindrical region. **b** Simulated energy-integrated X-ray image of the phantom (assuming no noise).

S7. Baggage Inspection Phantom Imaging

In addition to medical contrast imaging, X-ray imaging is commonly used for nondestructive testing in many different applications such as baggage inspection. We present below the material identification simulation results for a baggage inspection phantom where the 0.3 mm multicolor scintillator was compared to the ZnSe:Te and NaI:Tl single-layer scintillators of the same thicknesses, both using the clustering algorithm developed in this work. Our tests demonstrate that the multicolor scintillator reconstruction framework can be used for material identification in these applications and has superior performance compared to single-layer scintillators.

In baggage inspection, the objective is to distinguish between different items inside the bag. For material identification testing, we used the computational phantom shown in Fig. S16a. The thicknesses of each cylinder were determined such that energy-integrated imaging would show no difference in contrast between the cylinders (Fig. S17b) under a 67 kV tungsten X-ray source with a 0.3-cm Al filter (Fig. S18). This is to ensure that material identification is only possible through energy-resolution and not through differences in contrast. The effective linear attenuation coefficients in Fig. S17a were computed for each cylindrical region as:

$$\mu_{\text{eff}}(E) = \frac{1}{d}\int_{z=0}^{z=d} \mu(z, E)\mathrm{d}z$$

where z is the coordinate along the depth of the phantom, E is the energy, and d is the overall thickness of the phantom. Each substance included in the phantom represents different types of common day-to-day items: PET (plastic bottles, packaging, synthetic fiber clothes), HDPE (plastic bottles, packaging), muscle (meat products), water, cellulose (cotton clothes, plant-based products), glass, ceramic (dishes, cups), Al (laptops, touch pads, etc.), Fe (steel products), Au (jewelry). We assumed the ceramic to be 75% SiO_2 and 25% Al_2O_3.

As shown in Fig. S16b-c, the multicolor scintillator demonstrates superior performance compared to the ZnSe:Te and NaI:Tl single-layer scintillators. At low noise (Fig. S16d,

f), the multicolor scintillator can distinguish between inorganic (left-hand cylinders) and organic (right-hand cylinders) materials. Among the organic substances, it was able to distinguish between water-based (water, muscle), pure carbon-based (HDPE), and carbon-oxygen-based (PET, cellulose) materials. Among inorganic substances, oxides (glass, ceramic) could be distinguished from metals, two of which were individually identified (Fe and Au). At higher noise (Fig. S16h, j), the inorganic substances are no longer distinguishable from each other, but discrimination is still possible to a certain extent among the organic substances.

As can be seen from Fig. S17a, the materials in the baggage inspection phantom do not have any absorption edges between 16 and 67 keV. Therefore, in practice only 2 energy bins need to be measured accurately to distinguish between materials (i.e. the intermediate and high-energy bins correlate positively, so the intermediate bin need not be measured). ZnSe:Te, when combined with our postprocessing algorithm, is able to provide 2-bin energy resolution comparable to the multicolor scintillator, which is why their performances tend to be similar (Fig. S16b-c).

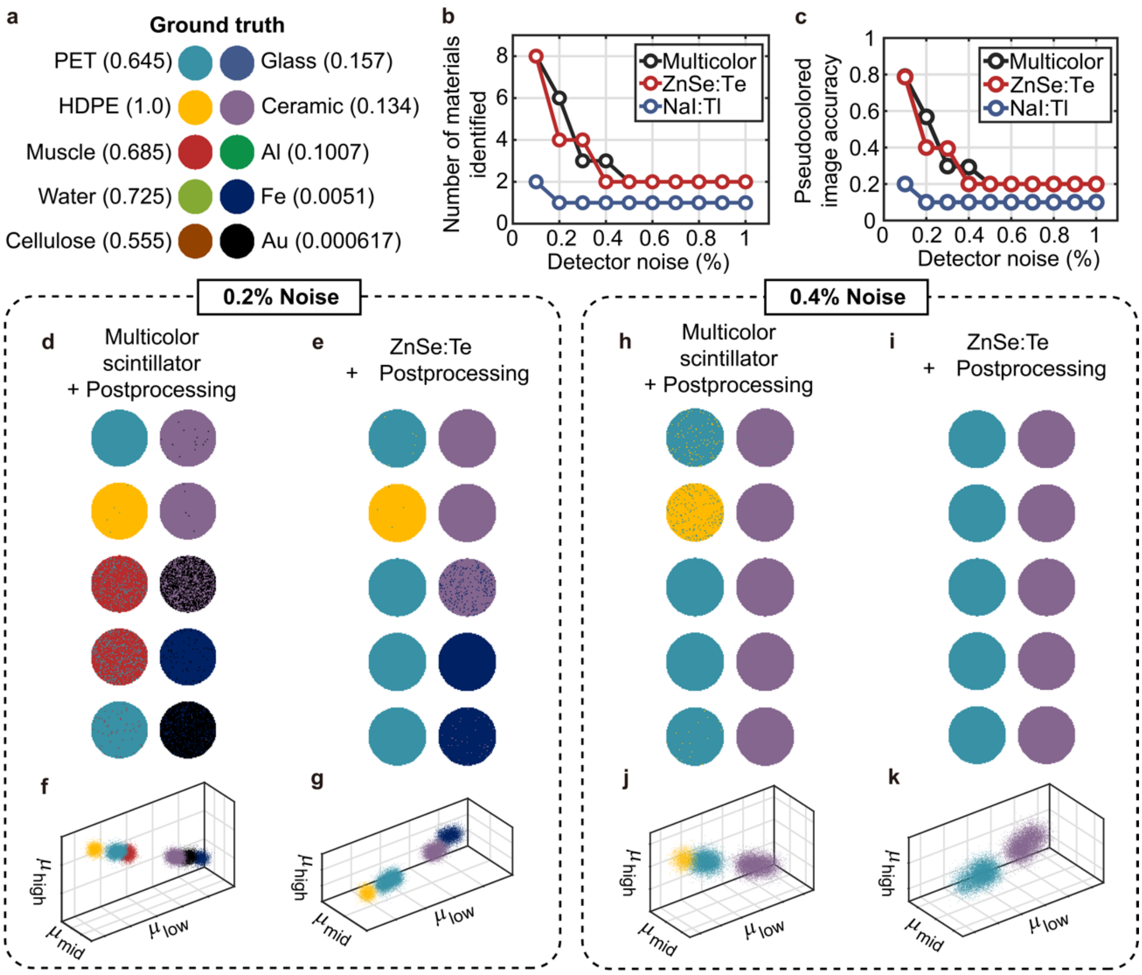

Figure S16. **Baggage inspection phantom simulation results. a** The ground truth computational phantom. The materials and heights (in cm) are listed beside each cylinder. The background is air. **b-c** The number of materials identified (**b**) and the accuracy of the pseudo-colored image (**c**) using different scintillators as a function of detector noise. **d-e** Pseudo-colored phantoms reconstructed using the multicolor and ZnSe:Te scintillators under 0.2% detector noise. **f-g** Energy maps for the multicolor and ZnSe:Te scintillators under 0.2% detector noise. **h-i** Pseudo-colored phantoms reconstructed using the multicolor and ZnSe:Te scintillators under 0.4% detector noise. **j-k** Energy maps for the multicolor and ZnSe:Te scintillators under 0.4% detector noise.

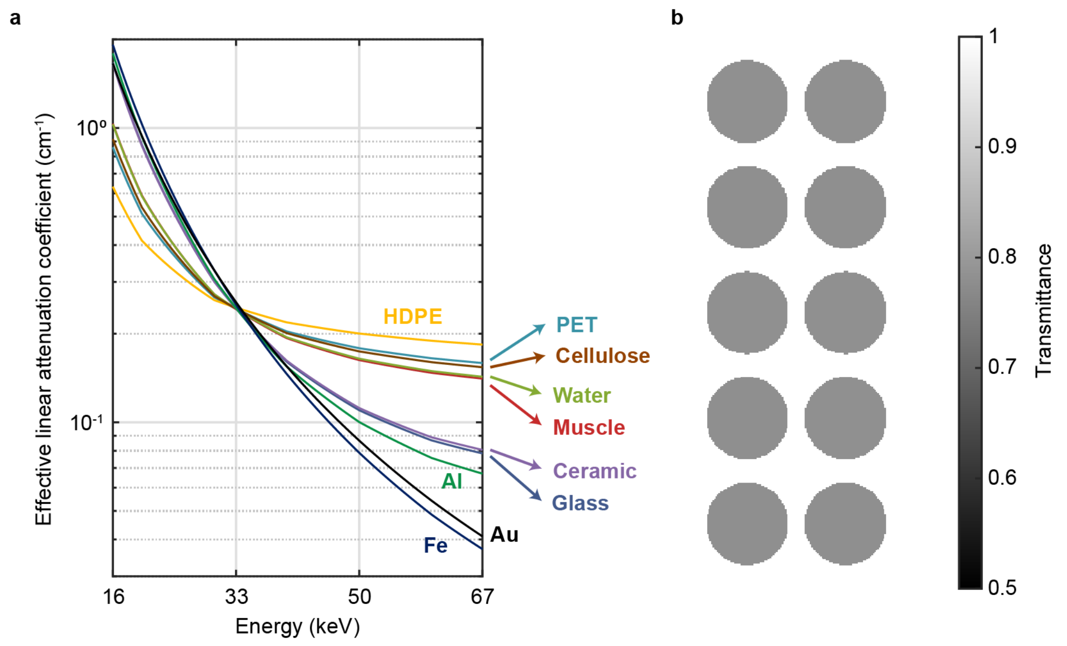

Figure S17. Simulated performance of energy-integrating detector. a The effective attenuation coefficient along the phantom depth in each cylindrical region. **b** Simulated energy-integrated X-ray image of the baggage phantom (assuming no noise).

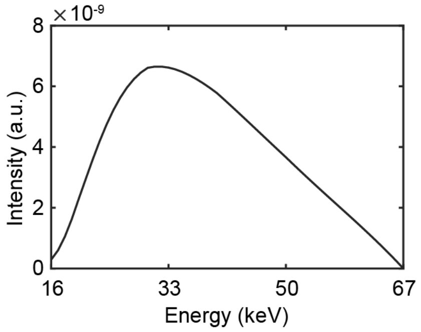

Figure S18. X-ray energy spectrum from a 67 kV tungsten source with a 0.3 cm-thick Al filter. Simulated using the model developed by Tucker *et al.*[2]. Characteristic K, L lines have been neglected for simplicity.

S8. Nanophotonic Angle Filter

In this section, we present additional details on the nanophotonic angle filter and its effects on the energy resolution of the multicolor scintillator reconstruction framework. Table S2 lists the optimized list of layer thicknesses for the angle filter and Fig. S19 shows the unpolarized reflectance of the angle filter in the direction from NaI:Tl to Gadox:Tb. Fig. S19 shows minimal reflection of upward-going scintillation photons from NaI:Tl, which prevents these photons from reaching the detector and enlarging the scintillation spots.

Table S2 Optimized nanophotonic angle filter. Layers are listed from top (Gadox:Tb side) to bottom (NaI:Tl side).

Index	Material	Thickness (nm)	Index	Material	Thickness (nm)
1	SiO_2	146.1	22	Si	80.0
2	Si	4.0	23	SiO_2	182.1
3	SiO_2	24.7	24	Si	11.2
4	Si	65.7	25	SiO_2	223.0
5	SiO_2	14.6	26	Si	9.9
6	Si	10.8	27	SiO_2	228.3
7	SiO_2	1280.2	28	Si	9.6
8	Si	6.1	29	SiO_2	213.7
9	SiO_2	45.8	30	Si	19.3
10	Si	7.4	31	SiO_2	214.9
11	SiO_2	212.3	32	Si	3.6
12	Si	3.5	33	SiO_2	2.7
13	SiO_2	250.7	34	Si	4.0
14	Si	3.6	35	SiO_2	4.3
15	SiO_2	198.0	36	Si	4.0
16	Si	63.3	37	SiO_2	3.0
17	SiO_2	11.4	38	Si	4.6
18	Si	13.2	39	SiO_2	224.0
19	SiO_2	196.2	40	Si	15.9
20	Si	8.1	41	SiO_2	215.0
21	SiO_2	199.0	42	Si	9.2

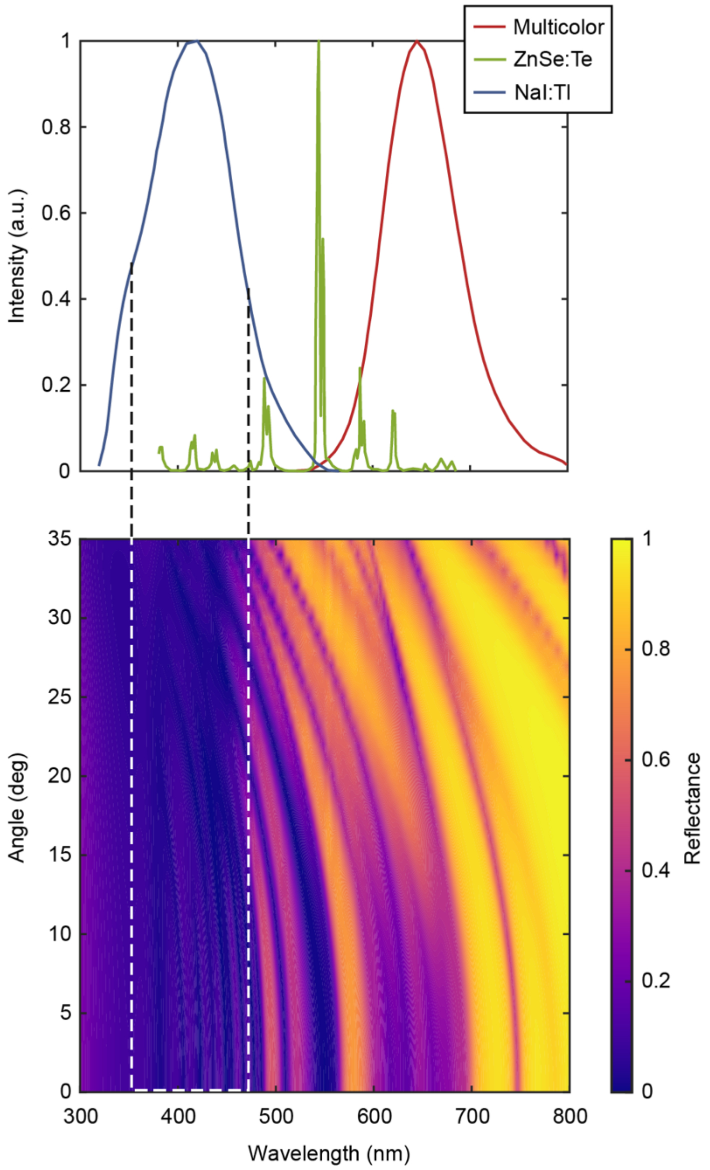

Figure S19 Unpolarized reflectance of the angle filter in the reverse direction. Photons with $\theta \gtrapprox 35°$ undergo TIR at the NaI:Tl-air interface.

Fig. S20 visually demonstrates the improvement of spatial resolution through the angle filter. Fig. S20a-b, d-e shows simulated images from a single frame of the RGB detector under different incident X-ray fluxes. The 0.3 mm-thick multicolor scintillator without the angle filter was used for these simulations. The detected photons have been grouped into clusters so that the lateral position and depth of the X-ray absorption could be identified (depth is indirectly identified through the dominant color and size of the cluster).

Different clusters are marked with different colors. It is clear that increasing X-ray flux increases the probability that scintillation occurs in nearby lateral positions, thereby causing increased overlap between the scintillation spots. This causes inaccurate clustering results, since our modified *k*-means algorithm does not allow overlapping clusters. By extension, this can also lead to inaccurate determination of the lateral position (and size) of the scintillation spots (Fig. S20h).

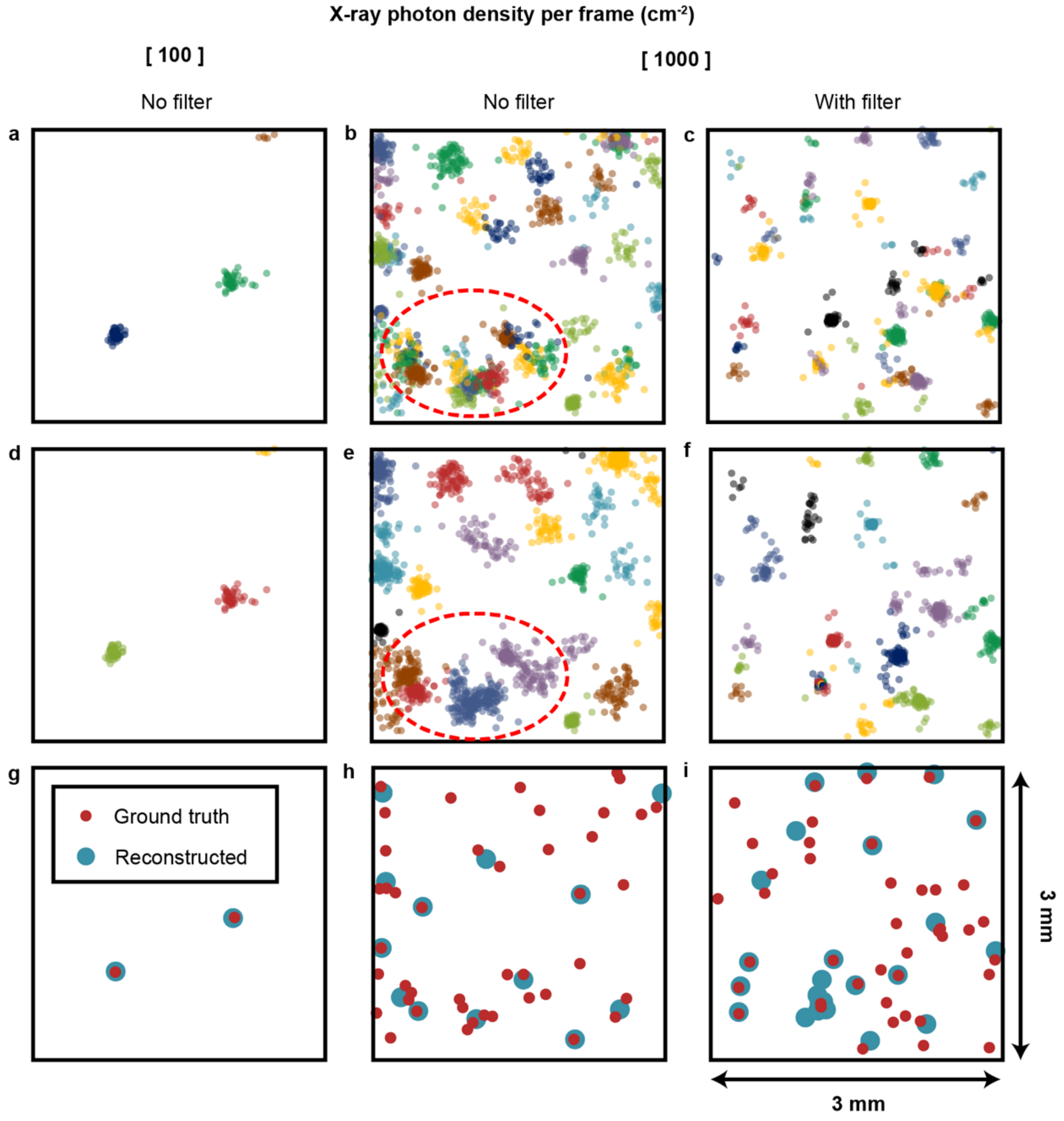

Figure S20. Single frame images of scintillation photons from the 0.3 mm-thick multicolor scintillator with (right) and without (left, center) the angular filter under X-ray photon densities of 100 (left) and 1000 (center, right) cm^{-2} per frame. **a-c** Ground truth images. Colors represent different photon clusters that each come from different

scintillation events. Some colors have been reused for several different clusters. Multiple clusters are overlapped inside the red dashed oval. **d-f** Reconstructed images. Colors represent reconstructed photon clusters. The multiple clusters inside the red dashed oval have been erroneously merged into fewer larger clusters. **g-i** Comparison of the centroid locations of the ground truth and reconstructed clusters.

The angle filter is able to reduce the spot sizes as shown in Fig. S20c. This especially improves the accuracy in the lateral positioning of the scintillation spots and allows the multicolor scintillator to work under higher X-ray flux without compromising spatial resolution.

Nevertheless, while it improves spatial resolution and maximum working X-ray flux density, the angle filter can negatively affect the energy resolution. The energy reconstruction accuracy of the 0.3 mm-thick multicolor scintillator with the filter is 42.3%, compared to 48.3% for the 0.3 mm-thick multicolor scintillator without the filter. The reduction in accuracy is due to the fact that scintillation events at the top and bottom of the scintillator have more similar spot sizes (i.e. cluster radii), which reduces the effectiveness of the spot size as an indirect measure of the absorption depth. Fig. S21 shows the medical phantom imaging results for the multicolor scintillator with and without filtering, where the degradation in material identification is apparent. Due to the fact that our postprocessing algorithm heavily relies on having a wide distribution of cluster radii to reconstruct the X-ray energy, there exists an unavoidable tradeoff relationship between spatial resolution (and X-ray flux density) and energy resolution. A similar well-known tradeoff also exists between spatial resolution and scintillation efficiency. Scintillation efficiency (proportion of incident X-ray that is absorbed by the scintillator) increases with the scintillator thickness, which in turn reduces the spatial resolution.

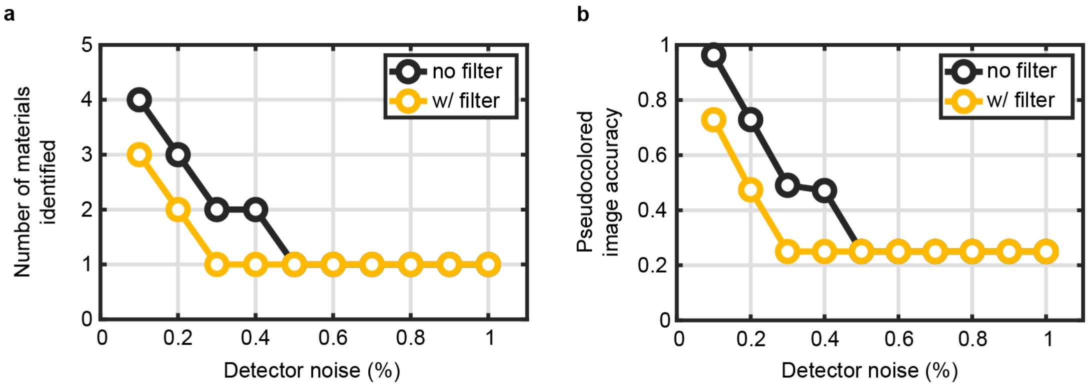

Figure S21. Medical phantom imaging results using the 0.3 mm-thick multicolor scintillator with and without the angular filter. **a** Number of materials identified as a function of the detector noise level. **b** Accuracy of the reconstructed pseudocolored images.

References

[1] W. Huda and R. B. Abrahams, “X-Ray-Based Medical Imaging and Resolution,” Am. J. Roentgenol. 204 (4), W393-W397 (2015).

[2] Tucker, D. M., Barnes, G. T., Chakraborty, D. P. Semiempirical model for generating tungsten target x-ray spectra. *Med. Phys.* **18**, 211-218 (1991).
